# One year of treatment with elexacaftor/tezacaftor/ivacaftor in patients with cystic fibrosis homozygous for the F508del mutation causes a significant increase in liver biochemical indexes

**DOI:** 10.3389/fmolb.2023.1327958

**Published:** 2024-01-08

**Authors:** Alice Castaldo, Monica Gelzo, Paola Iacotucci, Annalisa Longobardi, Giovanni Taccetti, Vito Terlizzi, Vincenzo Carnovale

**Affiliations:** ^1^ Dipartimento di Scienze Mediche Traslazionali, Centro Regionale Fibrosi Cistica del Bambino - Pediatria, Università di Napoli Federico II, Naples, Italy; ^2^ Ospedale Pediatrico Meyer IRCCS, Dipartimento di Scienze della Salute, Florence, Italy; ^3^ CEINGE-Biotecnologie Avanzate Franco Salvatore, Naples, Italy; ^4^ Dipartimento di Medicina Molecolare e Biotecnologie Mediche, Università di Napoli Federico II, Naples, Italy; ^5^ Dipartimento di Medicina Clinica e Chirurgia, Università di Napoli Federico II, Naples, Italy; ^6^ Meyer Children’s Hospital IRCCS, Centro Regionale Toscano per la cura della Fibrosi Cistica, Dipartimento di Pediatria, Firenze, Italy; ^7^ Dipartimento di Scienze Mediche Traslazionali, Centro Regionale Fibrosi Cistica dell’Adulto – Geriatria, Università di Napoli Federico II, Naples, Italy

**Keywords:** cystic fibrosis, lumacaftor/ivacaftor, elexacaftor/tezacaftor/ivacaftor, liver damage, cholesterol metabolism, children, pediatric patients

## Abstract

**Introduction:** Modulators of cystic fibrosis transmembrane conductance regulator mutated protein significantly improved the outcome of patients with cystic fibrosis (CF). We describe 63 patients who were independently followed up in two CF regional centers (i.e., Campania and Tuscany regions).

**Methods:** All patients were homozygous for the F508del mutation and were treated with lumacaftor/ivacaftor (LI) for 3 years, followed by 1 year of treatment with elexacaftor/tezacaftor/ivacaftor (ETI). We studied the biochemical parameters of liver damage and cholesterol metabolism.

**Results:** Beyond the improvement of BMI and lung function with LI treatment and even more with ETI, we found that the 3 years of LI treatment significantly improved liver function parameters (total and conjugated bilirubin, ALT, AP, and GGT), while the subsequent ETI treatment caused a significant increase of such parameters.

**Discussion:** We confirm that treatment with LI does not correct hypocholesterolemia, whereas treatment with ETI significantly increases serum cholesterol. Such an increase is likely due to enhanced *de novo* biosynthesis, as indicated by the significant increase in serum lathosterol, and it is likely that the subsequent liver cholesterol accumulation may contribute to triggering inflammation and worsening liver biochemical indexes. The increase in serum bilirubin and ALT that we observed in approximately 94% and 84% of patients treated with ETI, respectively, suggests further investigation of the impact of ETI therapy on liver function indexes.

## 1 Introduction

In the last decade, molecules that modulate the activity of cystic fibrosis transmembrane conductance regulator (CFTR) mutated proteins (modulators) have significantly improved the morbidity and outcome of patients with cystic fibrosis (CF). Single or various combinations of such drugs were used at the time, and the number of CF patients eligible for treatment has increased since the first studies. Only patients bearing a dozen *CFTR* mutations among the hundreds detected so far had access to molecular drugs. To date, both homozygous and heterozygous patients with the most frequent F508del mutation have become eligible for treatment. This process has been facilitated by *in vitro* and in *ex vivo* models, including organoids (Kleinfelder et al., 2023) and nasal epithelial cells (Di Lullo et al., 2017; Amato et al., 2019), that help to predict the responsivity to molecular drugs of each patient with CF. These models allowed for successful treatment of patients with either a rare (Terlizzi et al., 2021a) or single unknown *CFTR* mutation (Terlizzi et al., 2021b; Comegna et al., 2021).

Modulators enhance or restore the activity of CFTR through different mechanisms (Lopes-Pacheco, 2020). To date, four modulators have reached the market, namely, ivacaftor, which potentiates CFTR activity by enhancing the opening probability of the channel, lumacaftor, tezacaftor, and elexacaftor, which act as correctors of CFTR misfolding and misprocessing, enhancing the amount of protein at the membrane level. Such correctors recognize different binding sites of CFTR and may be combined (Lopes-Pacheco, 2020). Among the most effective protocols, there are the two-drug (i.e., lumacaftor/ivacaftor, LI) and three-drug (i.e., elexacaftor/tezacaftor/ivacaftor, ETI) combinations (Guo et al., 2022), the latter widely used to date (Bacalhau et al., 2023). Such protocol resulted in effective and safe randomized phase 3 studies (Heijerman et al., 2019) already after 24 weeks of treatment (Griese et al., 2021), and various studies described the improvement of sweat chloride levels, lung function (Zaher et al., 2021; Kapouni et al., 2023) and imaging indexes (Macconi et al., 2022), body composition, and exercise capacity (Gur et al., 2023) in patients with various *CFTR* genotypes, including those with at least one F508del allele (Middleton et al., 2019). In addition, our groups obtained effective improvement of sweat chloride and lung disease by ETI treatment in CF patients bearing the F508del mutation and a minimal function mutation (Carnovale et al., 2022a) and in patients homozygous for the F508del mutation (Carnovale et al., 2022b).

However, several studies reported a different degree of liver damage induced by ETI. A mild increase in transaminases in the first 3 months, not followed by a further increase (Tewkesbury et al., 2023) and an increase at 1 year that does not contraindicate the treatment (Wood et al., 2023), was reported. Furthermore, we observed hyperbilirubinemia, particularly in patients with *Gilbert disease*, in more than 10% of patients with CF after 3 months of treatment with ETI (Terlizzi et al., 2023). Sporadic cases of self-limited (Salehi et al., 2021; Stylemans et al., 2021) or severe liver injury and hepatic necrosis (Lowry et al., 2022; Bower et al., 2023) were also described. These reports contrast with studies describing only few cases of liver damage that contraindicate the continuation of ETI therapy (Zaher et al., 2021; Kapouni et al., 2023).

Furthermore, a severe reduction in serum cholesterol, mainly due to impaired absorption, was observed (Gelzo et al., 2016), particularly in patients with pancreatic insufficiency (Gelzo et al., 2020). Such a condition induces an increase in *de novo* synthesis of cholesterol that does not correct hypocholesterolemia because the alteration in CFTR reduces the blood release of cholesterol, causing the accumulation of liver cholesterol that contributes to inflammation, as observed in the CF mouse model (Amato et al., 2021). The use of LI improves liver cholesterol metabolism but does not correct hypocholesterolemia (Gelzo et al., 2021). No data are available on cholesterol metabolism in patients with CF treated with ETI.

Thus, in the present study, we describe patients with CF who were independently followed up in two CF regional centers (i.e., Campania and Tuscany regions); all patients were homozygous for the F508del mutation and were treated for 3 years with LI, followed by 1 year of treatment with ETI. In order to assess the impact of these treatments on liver function and cholesterol metabolism, we studied biochemical parameters of liver damage and the metabolism of cholesterol, including surrogate markers of cholesterol absorption and *de novo* synthesis.

## 2 Materials and methods

### 2.1 Patients

The study was approved by the ethics committee of the CF regional center of Tuscany (Florence, Comitato Etico, number 312/2022) and the CF regional center of Campania (Naples, Comitato Etico Federico II, number 77/2021). Informed consent was obtained from all patients (or from their legal guardians) for the use of anonymous clinical data for research purposes. Criteria for entering the study included homozygosity for the F508del mutation, together with treatment for at least 3 years with LI, followed by at least 1 year of treatment with ETI. Exclusion criteria included mechanical ventilation, CF liver disease (CFLD), history of solid organ or hematological transplantation, history of drug or alcohol abuse in the past year, and pregnancy (TRIKAFTA prescribing information, 2020).

We studied 63 patients with CF, all homozygous for the F508del mutation. Among these patients, 33 were recruited at the regional center of Campania and 30 at the regional center of Tuscany (Table 1). No significant difference was observed between the two populations for age and gender. Both clinical centers shared the procedures for the treatment and follow-up of the patients according to national standards for CF care. In particular, during the whole period of the study, all patients were followed up by a dietician, and their diet and support therapy (including integrators and enzyme supplementation) did not change. All patients enrolled in the study performed a sequencing of all *CFTR* exons (Bergougnoux et al., 2018) to exclude other mutations in addition to F508del and an analysis of large gene rearrangements to confirm the homozygosity for F508del (Tomaiuolo et al., 2008). Forced expiratory volume (FEV_1_) was expressed as the percentage of the predicted value for age, according to standardized reference equations for spirometry (Terlizzi et al., 2018). Liver disease was evaluated by clinical, biochemical, or ultrasonography abnormalities (Bartlett et al., 2009) recorded in two consecutive examinations within a 3-month period in the absence of other causes of congenital or acquired chronic liver disease. Among the 63 patients enrolled in the study, 27 patients (13/30 from Tuscany and 14/33 from Campania) had liver steatosis at baseline (and after the two treatments). No other cases of steatosis or other liver alterations were evidenced at the end of the two treatments (i.e., 3 years of LI followed by 1 year of ETI). The body mass index was evaluated as previously described (Elce et al., 2018). ETI treatment was administered orally according to the manufacturer’s recommendations (200 mg elexacaftor/100 mg tezacaftor/150 mg ivacaftor in the morning and 150 mg ivacaftor in the evening).

**TABLE 1:** Demographic data of 63 CF patients homozygous for F508del from Campania and Tuscany regions.

	Campania	Tuscany
*n*	33	30
Age (years)^a^	24 (20–30)	24 (17–32)
Gender, male (%)	10 (30)	15 (50)

^a^ Median (interquartile range).

### 2.2 Biochemical parameters

Glucose, total cholesterol, high-density lipoprotein (HDL) and low-density lipoprotein (LDL) cholesterol, triglycerides, total and conjugated bilirubin, alkaline phosphatase (AP), alanine aminotransferase (ALT), gamma glutamyl transferase (GGT), and albumin were evaluated on serum within 1 hour from the blood sampling by using automated analyzers using standard procedures. For all patients, the samples collected at the different times were analyzed in the same laboratory. The analysis of plasma lathosterol, as a *de novo* synthesis surrogate marker, and cholestanol, as a marker of intestinal absorption efficiency (Nikkila et al., 2005; Nikkila et al., 2008), was performed by gas chromatography as previously described (Gelzo et al., 2016; Gelzo et al., 2021).

### 2.3 Statistical analysis

Continuous data were reported as median and IQR. The Shapiro–Wilk test was applied to evaluate the normality of distributions. Paired comparisons among the four time points were evaluated using Friedman’s test. Statistical analyses were performed by SPSS (version 29, IBM SPSS Statistics). Graphics were carried out using KaleidaGraph software (version 4.5.4, Synergy, Reading, PA, United States). *p*-values < 0.05 were considered significant.

## 3 Results

Table 2 shows the comparison of the anthropometric and biochemical parameters obtained from 33 patients with CF homozygous for F508del; these patients were followed up in the regional center of Campania at different stages: before any treatment (baseline), after 1 year of treatment with LI, after 3 years of treatment with LI, and after a subsequent year of treatment with ETI. The values of body mass index significantly increased after 1 year of treatment with LI, as compared to baseline, and remained stable after 3 years of treatment. The subsequent treatment with ETI caused a further significant increase in body mass index as compared to the treatment with LI. FEV_1_ showed an increasing trend after 1 year of treatment with LI and remained stable after 3 years of treatment. The values significantly increased after 1 year of treatment with ETI, both in comparison to baseline values and to the values obtained after treatment with LI.

**TABLE 2:** Comparison of anthropometric and biochemical parameters in 33 CF patients homozygous for F508del from the Campania region at baseline and after lumacaftor/ivacaftor (LI) and elexacaftor/tezacaftor/ivacaftor (ETI) therapies.

	r.v.	Baseline	1 year of LI	3 years of LI	1 year of ETI
Body mass index (kg/m^2^)		21 (20–22)	22 (20–23)^a^	22 (20–23)^a^	23 (22–25)^a^ ^,^ ^b^ ^,^ ^c^
FEV_1_ (%)	>80	48 (37–76)	53 (38–81)	54 (33–83)	66 (46–98)^a^ ^,^ ^b^ ^,^ ^c^
Glucose (mg/dL)	70–110	82 (77–97)	80 (72–96)	81 (72–91)	78 (71–92)
Total cholesterol (mg/dL)	121–232	109 (98–136)	117 (109–130)	125 (107–138)	148 (129–159)^a^ ^,^ ^b^ ^,^ ^c^
HDL cholesterol (mg/dL)	>40	46 (35–54)	51 (42–57)	50 (43–56)	54 (44–63)^a^ ^,^ ^c^
LDL cholesterol (mg/dL)	<115	58 (53–70)	64 (50–74)	57 (49–73)	80 (62–95)^a^ ^,^ ^b^ ^,^ ^c^
Triglycerides (mg/dL)	<150	67 (55–74)	61 (51–72)	60 (54–75)	66 (59–81)
Liver parameters					
Alkaline phosphatase (U/L)	40–150	109 (96–117)	68 (58–86)^a^	67 (58–92*)* ^a^	113 (89–124)^b^ ^,^ ^c^
GGT (U/L)	12–64	14 (11–21)	11 (10–21)^a^	12 (10–19)^a^	19 (14–34)^b^ ^,^ ^c^
Total bil (mg/dL)	0.2–1.2	0.55 (0.40–0.84)	0.36 (0.27–0.44)^a^	0.36 (0.27–0.48)^a^	1.03 (0.59–1.52)^a^ ^,^ ^b^ ^,^ ^c^
Conjugated bil (mg/dL)	0–0.40	0.29 (0.20–0.38)	0.17 (0.15–0.25)^a^	0.20 (0.15–0.24)^a^	0.39 (0.27–0.59)^a^ ^,^ ^b^ ^,^ ^c^
ALT (U/L)	0–55	24 (17–29)	19 (14–29)^a^	18 (12–32)	42 (24–55)^a^ ^,^ ^b^ ^,^ ^c^
Albumin (g/dL)	3.5–5.2	4.3 (4.2–4.5)	4.5 (4.3–4.8)^a^	4.5 (4.3–4.8)^a^	4.5 (4.3–4.8)^a^

ALT, alanine aminotransferase; bil, bilirubin; FEV, forced expiratory volume; GGT, gammaglutamyltransferase; HDL, high-density lipoprotein; LDL, low-density lipoprotein; r.v., reference values.

Data are reported as median (IQR).

^a^ *p* < 0.05 vs. baseline.

^b^ *p* < 0.05 vs. 1 year LI.

^c^ *p* < 0.05 vs. 3 years LI.

Serum glucose and triglycerides were not significantly modified by any of the treatments. Serum AP and serum GGT were significantly reduced after 1 year of treatment with LI, and their values remained stable after 3 years of treatment. After 1 year of treatment with ETI, serum values of both biomarkers significantly increased as compared to 1 year and 3 years of LI treatment. Serum total bilirubin and conjugated bilirubin were significantly reduced after 1 year of treatment with LI as compared to baseline values, while 1 year of treatment with ETI caused a significant increase in both the biomarkers as compared both to baseline values and the values obtained after 1 year and 3 years of treatment with ETI. Serum ALT mirrored the trend of bilirubin, with a significant reduction after 1 year of LI treatment, which was maintained after 3 years of LI treatment, and a significant increase after 1 year of ETI. Serum albumin significantly increased after 1 year of treatment with LI, and the values remained stable after 3 years of treatment with LI and after 1 year with ETI. Serum total cholesterol and HDL and LDL cholesterol were not significantly changed by the treatment with LI, while they significantly increased after 1 year of treatment with ETI, as compared to both baseline values and values obtained after the treatment with LI.

The same comparisons were performed on the 30 patients with CF followed in the regional center of Tuscany (Table 3). The data obtained confirmed all the results obtained in the patients from the center of Campania. The trend in BMI and FEV_1_ was comparable to that observed in the first group of patients, as was the trend in biochemical parameters of liver function (i.e., AP, GGT, total and conjugated bilirubin, ALT, and albumin) and lipid metabolism (i.e., total, HDL, and LDL cholesterol and triglycerides).

**TABLE 3:** Comparison of anthropometric and biochemical parameters in 30 CF patients homozygous for F508del from the Tuscany region at baseline and after lumacaftor/ivacaftor (LI) and elexacaftor/tezacaftor/ivacaftor (ETI) therapies.

	r.v.	Baseline	1 year of LI	3 years of LI	1 year of ETI
Body mass index (kg/m^2^)		20 (18–21)	21 (19–23)^a^	22 (20–23)^a^	22 (21–24)^a^ ^,^ ^b^
FEV_1_ (%)	>80	62 (41–77)	68 (53–86)^a^	65 (45–86)	75 (58–95)^a^ ^,^ ^c^
Glucose (mg/dL)	70–110	86 (78–94)	88 (82–101)	91 (84–107)	88 (82–100)
Total cholesterol (mg/dL)	121–232	121 (95–165)	121 (104–152)	129 (111–162)	144 (138–174)^a^ ^,^ ^b^ ^,^ ^c^
HDL cholesterol (mg/dL)	>40	46 (39–55)	48 (37–56)	50 (40–58)	53 (45–57)
LDL cholesterol (mg/dL)	<115	56 (43–84)	52 (45–84)	61 (47–86)	86 (71–102)^a^ ^,^ ^b^ ^,^ ^c^
Triglycerides (mg/dL)	<150	77 (58–127)	78 (63–97)	75 (59–103)	95 (64–110)
Liver parameters					
Alkaline phosphatase (U/L)	40–150	118 (73–181)	78 (59–115)^a^	87 (65–108)^a^	113 (90–133)^b^ ^,^ ^c^
GGT (U/L)	12–64	13 (8–18)	13 (7–15)	11 (8–15)	20 (16–23)^a^ ^,^ ^b^ ^,^ ^c^
Total bil (mg/dL)	0.2–1.2	0.35 (0.30–0.42)	0.30 (0.20–0.40)	0.30 (0.20–0.40)	0.80 (0.60–1.1)^a^ ^,^ ^b^ ^,^ ^c^
Conjugated bil (mg/dL)	0–0.40	0.10 (0.10–0.10)	0.10 (0.10–0.10)	0.10 (0.10–0.20)	0.30 (0.20–0.40)^a^ ^,^ ^b^ ^,^ ^c^
ALT (U/L)	0–55	27 (21–39)	27 (20–44)	22 (18–29)^a^	32 (24–45)^c^
Albumin (g/dL)	3.5–5.2	5.3 (5.0–5.7)	5.6 (5.3–6.0)^a^	5.5 (5.2–6.1)^a^	5.9 (5.7–6.0)^a^

ALT, alanine aminotransferase; bil, bilirubin; FEV, forced expiratory volume; GGT, gammaglutamyltransferase; HDL, high-density lipoprotein; LDL, low-density lipoprotein; r. v., reference values.

Data are reported as median (IQR).

^a^ *p* < 0.05 vs. baseline.

^b^ *p* < 0.05 vs. 1 year LI.

^c^ *p* < 0.05 vs. 3 years LI.

**FIGURE 1:**
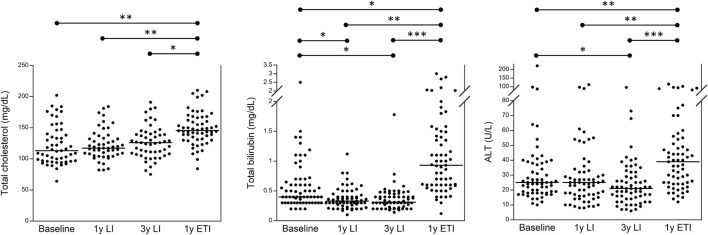
Serum levels of total cholesterol, total bilirubin, and ALT in 63 patients with CF homozygous for F508del followed in the regional centers of Campania and Tuscany before any treatment (baseline), after 1 year of treatment with LI, after 3 years of treatment with LI, and after a subsequent year of treatment with ETI. *p < 0.05, **p < 0.005, and ***p < 0.0005.

Figure 1 and Supplementary Table S1 group all 63 patients with CF and show that the treatment with ETI increased serum values of biochemical parameters related to liver function in most patients with CF. Specifically, 59/63 (93.7%) patients experienced an increase in serum total bilirubin, and 53/63 (84.1%) had an increase in serum ALT as compared to the values after 3 years of treatment with LI. However, in none of the 63 patients, we recorded an increase in serum values three times higher than the upper reference value (i.e., 1.2 mg/dL for total bilirubin and 55 U/L for serum ALT). Supplementary Table S2 shows the number of patients with lab test values above the reference value at baseline, after 3 years of treatment with LI, and after a further year of treatment with ETI.

**FIGURE 2:**
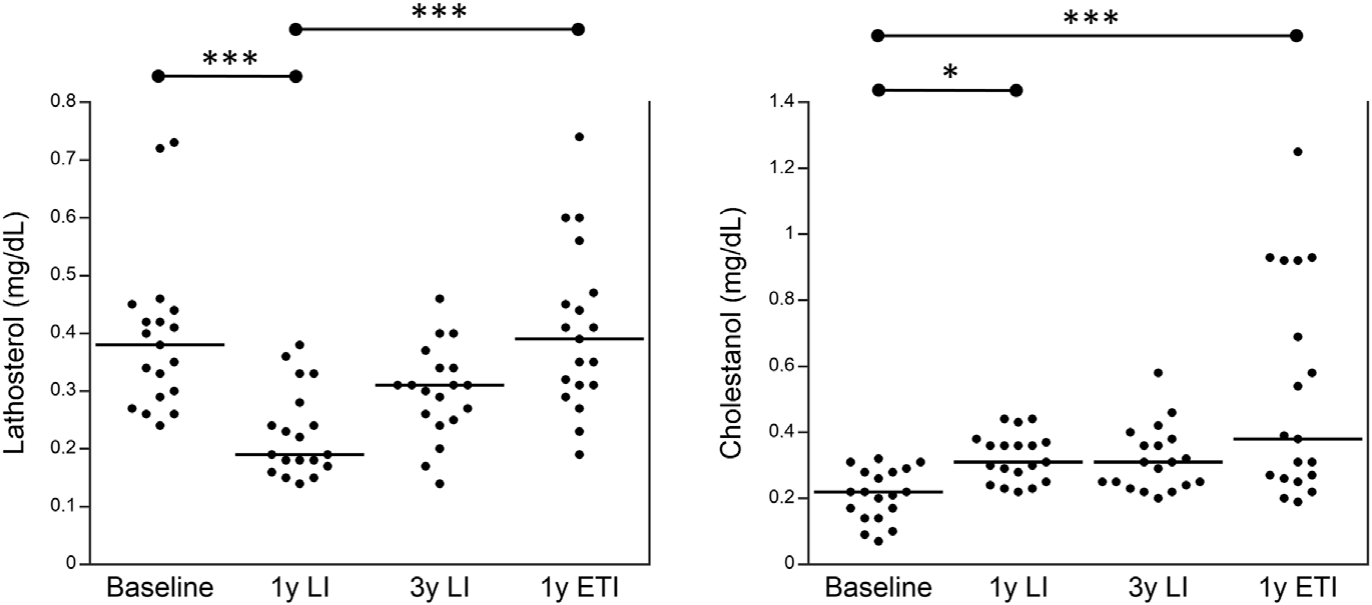
Plasma levels of lathosterol and cholestanol in 19 patients with CF homozygous for F508del followed in the regional center of Campania before any treatment (baseline), after 1 year of treatment with LI, after 3 years of treatment with LI, and after a subsequent year of treatment with ETI. *p < 0.05 and ***p < 0.0005.

Finally, Figure 2 shows the values of plasma sterols obtained from 19 patients with CF homozygous for F508del; these patients were followed up in the regional center of Campania at different stages: before any treatment (baseline), after 1 year of treatment with LI, after 3 years of treatment with LI, and after a subsequent year of treatment with ETI. Plasma lathosterol was significantly reduced after 1 year of treatment with LI as compared to baseline, whereas it significantly increased after 1 year of treatment with ETI as compared to the values obtained after 1 year of treatment with LI. The levels of plasma cholestanol significantly increased after 1 year of treatment with LI and further increased after 1 year of treatment with ETI as compared to baseline.

## 4 Discussion

The data from the present study, obtained from two independent populations of patients with CF homozygous for the F508del mutation followed up in two different regional centers, indicate that 3 years of treatment with LI significantly improved BMI, and one following year of treatment with ETI further improved, significantly, such a parameter. ETI treatment also displayed an effect on lung function, significantly enhancing FEV_1_. The 3 years of treatment with LI improved serum biochemical parameters of liver function (i.e., significant reduction of total and conjugated bilirubin, ALT, AP, and GGT), whereas the subsequent treatment for 1 year with ETI caused a significant increase in these parameters compared to the data obtained after the 3 years of LI treatment and basal data before any treatment. We also compared the median values of these biochemical liver parameters in two subgroups of patients, i.e., 27 CF patients with steatosis and 36 without steatosis, at baseline and at the end of the treatment, and no significant differences were observed. In addition, the treatment with LI does not correct hypocholesterolemia, as we previously observed in a preliminary study (Gelzo et al., 2021), whereas the treatment with ETI significantly increased total serum cholesterol and LDL cholesterol. These findings agree with a study on 41 patients with CF that relates the increase in total serum cholesterol and LDL cholesterol with CF-related liver disease (Despotes et al., 2023).

Overall, these data confirm the positive effect of LI on liver function that was previously described in a study on 37 adolescents homozygous for the F508del mutation (Drummond et al., 2022) and in another study that reported the improvement of liver function tests, including biomarkers of liver fibrosis, in 39 patients with CF treated with LI, suggesting such therapy as a potential approach to reverting liver fibrosis in patients with CF (Levitte et al., 2023). On the other hand, various trials reported that the therapy with LI is associated with an increase in liver enzymes in a percentage of cases not significantly different from that reported in placebo groups (Wainwright et al., 2015), and a revision does not report cases of severe liver complications in patients with CF treated with LI (Lopes-Pacheco, 2020). The positive effect of LI on liver function may be related to the improvement of biliary salt metabolism (Levitte et al., 2023). On the other hand, ETI therapy significantly improved BMI and FEV_1_ in agreement with all previous studies (Heijerman et al., 2019; Middleton et al., 2019; Griese et al., 2021; Zaher et al., 2021; Macconi et al., 2022; Kapouni et al., 2023), but it significantly worsens the liver metabolism. In fact, 1 year of treatment with ETI caused an enhancement in liver necrosis, with a significant increase in ALT already reported in several studies (Salehi et al., 2021; Stylemans et al., 2021; Lowry et al., 2022; Bower et al., 2023; Tewkesbury et al., 2023; Wood et al., 2023). Furthermore, we observed an increase in AP and GGT, suggesting the involvement of the intrahepatic biliary tree and the impairment of bile metabolism. This is also suggested by the significant increase in serum bilirubin that we previously observed in a preliminary group of CF patients treated with ETI (Terlizzi et al., 2023) and by the abnormal increase in plasma cholestanol in 26% of patients after 1 year of ETI treatment. Comparable levels of circulating cholestanol are observed in biliary cirrhosis and other cholestatic diseases, with cholestanol being a sensitive marker of cholestasis (Nikkila et al., 2005; Nikkila et al., 2008). The increase in both total and conjugated serum bilirubin may depend on the bile loss at the intestinal level with enhanced reabsorption of conjugated bilirubin or, more likely, on the inhibition of OATP1B1 and 1B3 by ETI (Terlizzi et al., 2023). However, among the effects of ETI, we observed a significant increase in total serum cholesterol that is likely due to both enhanced intestinal absorption efficiency and *de novo* biosynthesis, as indicated by the significant increase in serum cholestanol and lathosterol, respectively. In a study on the CF mouse model, we demonstrated that the enhanced *de novo* synthesis of cholesterol causes intracellular accumulation of cholesterol at the endosome level (Amato et al., 2021) as a consequence of the misassembled F508del CFTR protein (Gentzsch et al., 2007). The accumulation of liver cholesterol triggers inflammation (Amato et al., 2021) and may contribute to the worsening of liver function that we observed after ETI treatment.

In conclusion, the present study includes a large number of patients with CF with a homogeneous CFTR genotype recruited and studied independently in two regional CF centers. The main limitation of the study is that the patients performed a single year of treatment with ETI, since the use of such a combination was recently approved for the treatment of patients with CF homozygous for the F508del mutation. The increase in serum bilirubin and ALT that we observed in approximately 94% and 84% of patients, respectively (although no patient had values three times higher than the upper reference limit and no patient developed any clinical or instrumental evidence of CFLD), suggests further investigation of the impact of the ETI therapy on liver function indexes.

## Data Availability

The original contributions presented in the study are included in the article/Supplementary Material; further inquiries can be directed to the corresponding author.
