# Supplementary material for: One year of treatment with elexacaftor/tezacaftor/ivacaftor in patients with cystic fibrosis homozygous for the F508del mutation causes a significant increase in liver biochemical indexes
Source: Front Mol Biosci. 2024 Jan 8;10:1327958. doi: 10.3389/fmolb.2023.1327958 (PMC10800484; doi:10.3389/fmolb.2023.1327958)
Supplement: Supplementary file 1 [file Table1.docx]

Supplementary Material

**Supplementary Table 1.** Liver biochemical parameters in 63 patients with CF at baseline, after 1 year of therapy with LI, after 3 years of therapy with LI and after one subsequent year of therapy with ETI.

|  | **Baseline** | | **After 1 year of LI** | | **After 3 years of LI** | | **After 1 year of ETI** | |
| --- | --- | --- | --- | --- | --- | --- | --- | --- |
| **ID** | **Total Bil** | **ALT** | **Total Bil** | **ALT** | **Total Bil** | **ALT** | **Total Bil** | **ALT** |
| Tk84 | 0.4 | 18 | 0.4 | 36 | 0.3 | 23 | 0.4 | 18 |
| TK22 | 0.6 | 17 | 0.3 | 18 | 0.5 | 18 | 1.4 | 55 |
| TK23 | 0.6 | 25 | 0.2 | 10 | 0.3 | 10 | 1.6 | 44 |
| TK25 | 0.2 | 17 | 0.4 | 34 | 0.2 | 10 | 0.6 | 21 |
| TK28 | 0.6 | 98 | 0.5 | 30 | 0.5 | 35 | 1.5 | 56 |
| TK32 | 1.5 | 87 | 1.1 | 32 | 1.8 | 32 | 2.2 | 48 |
| TK38 | 1.4 | 31 | 0.8 | 29 | 0.5 | 26 | 2.1 | 82 |
| TK40 | 0.4 | 12 | 0.3 | 8 | 0.2 | 12 | 0.4 | 40 |
| TK41 | 0.4 | 15 | 0.5 | 12 | 0.5 | 12 | 1.8 | 25 |
| TK42 | 0.2 | 24 | 0.2 | 14 | 0.2 | 14 | 0.6 | 44 |
| TK44 | 1.1 | 19 | 0.3 | 12 | 0.3 | 16 | 2 | 70 |
| TK50 | 0.5 | 14 | 0.4 | 17 | 0.2 | 11 | 1 | 18 |
| TK54 | 0.7 | 25 | 0.3 | 23 | 0.3 | 36 | 0.5 | 12 |
| TK62 | 0.4 | 16 | 0.3 | 10 | 0.3 | 10 | 1.3 | 49 |
| TK63 | 1.1 | 38 | 0.6 | 33 | 0.6 | 39 | 1.2 | 75 |
| TK70 | 0.6 | 20 | 0.3 | 19 | 0.4 | 14 | 2 | 115 |
| TK71 | 0.7 | 19 | 0.4 | 25 | 0.5 | 22 | 1.5 | 34 |
| TK73 | 0.5 | 18 | 0.3 | 14 | 0.2 | 15 | 0.9 | 37 |
| TK77 | 0.3 | 25 | 0.4 | 24 | 0.3 | 21 | 0.6 | 94 |
| TK78 | 0.7 | 22 | 0.4 | 15 | 0.3 | 28 | 1.6 | 58 |
| TK24 | 0.5 | 11 | 0.3 | 8 | 0.3 | 7 | 0.9 | 14 |
| TK29 | 0.4 | 14 | 0.3 | 9 | 0.4 | 7 | 0.9 | 25 |
| TK30 | 1.1 | 221 | 0.4 | 54 | 0.4 | 21 | 0.5 | 77 |
| TK31 | 0.7 | 27 | 0.2 | 18 | 0.5 | 12 | 1.4 | 19 |
| TK33 | 0.5 | 25 | 0.4 | 17 | 0.7 | 41 | 0.1 | 18 |
| TK34 | 0.5 | 17 | 0.4 | 52 | 0.3 | 12 | 1.5 | 40 |
| TK35 | 1 | 64 | 0.2 | 14 | 0.4 | 16 | 0.7 | 42 |
| Tk56 | 0.6 | 25 | 0.4 | 22 | 0.4 | 32 | 0.4 | 25 |
| Tk58 | 0.4 | 24 | 0.2 | 23 | 0.1 | 16 | 0.6 | 47 |
| Tk64 | 1.2 | 25 | 0.6 | 16 | 0.8 | 42 | 1.4 | 39 |
| Tk76 | 0.6 | 40 | 0.5 | 25 | 0.5 | 96 | 0.9 | 51 |
| Tk81 | 0.5 | 26 | 0.3 | 54 | 0.2 | 37 | 0.6 | 54 |
| Tk83 | 1 | 39 | 0.6 | 25 | 0.5 | 22 | 1.4 | 24 |
| T1 | 1.3 | 41 | 0.6 | 55 | 0.5 | 36 | 3 | 45 |
| T2 | 0.4 | 49 | 0.2 | 97 | 0.3 | 68 | 1 | 32 |
| T3 | 0.3 | 18 | 0.2 | 17 | 0.2 | 7 | 0.5 | 22 |
| T4 | 0.3 | 22 | 0.3 | 39 | 0.2 | 19 | 0.7 | 36 |
| T5 | 1.4 | 26 | 0.8 | 24 | 0.4 | 9 | 2.7 | 30 |
| T6 | 0.4 | 18 | 0.4 | 10 | 0.3 | 9 | 1.1 | 24 |
| T7 | 0.2 | 27 | 0.3 | 53 | 0.3 | 40 | 0.3 | 26 |
| T8 | 0.3 | 40 | 0.4 | 25 | 0.2 | 25 | 0.6 | 40 |
| T9 | 0.3 | 27 | 0.3 | 29 | 0.2 | 19 | 0.5 | 24 |
| T10 | 0.4 | 34 | 0.3 | 59 | 0.4 | 29 | 0.7 | 42 |
| T11 | 0.4 | 24 | 0.4 | 27 | 0.3 | 17 | 0.4 | 16 |
| T12 | 0.4 | 23 | 0.3 | 20 | 0.2 | 21 | 1.1 | 102 |
| T13 | 0.9 | 37 | 0.3 | 90 | 0.3 | 17 | 1.8 | 36 |
| T14 | 2.5 | 32 | 0.7 | 28 | 0.5 | 22 | 2.8 | 60 |
| T15 | 0.3 | 43 | 0.2 | 32 | 0.3 | 25 | 1.1 | 89 |
| T16 | 0.2 | 19 | 0.2 | 18 | 0.2 | 19 | 0.6 | 21 |
| T17 | 0.7 | 54 | 0.5 | 25 | 0.4 | 17 | 1.9 | 22 |
| T18 | 0.3 | 23 | 0.3 | 9 | 0.3 | 25 | 0.9 | 44 |
| T19 | 0.4 | 20 | 0.3 | 20 | 0.4 | 21 | 0.6 | 30 |
| T20 | 0.3 | 28 | 0.3 | 112 | 0.4 | 33 | 1 | 43 |
| T21 | 0.3 | 17 | 0.5 | 27 | 0.3 | 26 | 0.8 | 33 |
| T22 | 0.4 | 43 | 0.2 | 61 | 0.5 | 19 | 0.7 | 20 |
| T23 | 0.3 | 24 | 0.1 | 16 | 0.2 | 22 | 0.5 | 28 |
| T24 | 0.3 | 63 | 0.2 | 26 | 0.2 | 73 | 0.6 | 47 |
| T25 | 0.6 | 22 | 0.5 | 41 | 0.4 | 19 | 1.1 | 29 |
| T26 | 0.5 | 39 | 0.4 | 27 | 0.3 | 29 | 1 | 92 |
| T27 | 0.3 | 18 | 0.3 | 23 | 0.3 | 49 | 2 | 96 |
| T28 | 0.4 | 38 | 0.3 | 27 | 0.2 | 26 | 0.8 | 70 |
| T29 | 0.3 | 10 | 0.2 | 9 | 0.4 | 6 | 0.6 | 15 |
| T30 | 0.3 | 30 | 0.3 | 27 | 0.2 | 22 | 0.4 | 29 |

LI: Lu-macaftor/Ivacaftor; ETI: Elexacaftor/Tezacaftor/Ivacaftor; Total Bil: total bilirubin; ALT: alanine aminotransferase.
