# Supplementary material for: One year of treatment with elexacaftor/tezacaftor/ivacaftor in patients with cystic fibrosis homozygous for the F508del mutation causes a significant increase in liver biochemical indexes
Source: Front Mol Biosci. 2024 Jan 8;10:1327958. doi: 10.3389/fmolb.2023.1327958 (PMC10800484; doi:10.3389/fmolb.2023.1327958)
Supplement: Supplementary file 2 [file Table2.docx]

Supplementary Material

**Supplementary Table 2.** Number of patients with lab test values above the reference value at baseline, after 3 years of treatment with LI and after a further year of treatment with ETI

|  | **Baseline** | **After 3 years of LI** | **After 1 year of ETI** |
| --- | --- | --- | --- |
| Alkaline phosphatase | 9 | 2 | 5 |
| GGT | 2 | 0 | 2 |
| Total bilirubin | 3 | 0 | 5 |
| Conjugated bilirubin | 0 | 0 | 6 |
| ALT | 1 | 2 | 6 |
| Albumin | 0 | 0 | 0 |
